# An All-Solid-State Silicate Ion-Selective Electrode Using PbSiO_3_ as a Sensitive Membrane

**DOI:** 10.3390/s19030525

**Published:** 2019-01-27

**Authors:** Rongrong Wu, Xue-Gang Chen, Chunhui Tao, Yuanfeng Huang, Ying Ye, Qiujin Wang, Yifan Zhou, Quan Jin, Wei Cai

**Affiliations:** 1 Ocean College, Zhejiang University, Zhoushan 316021, China; 18358087818@163.com (R.W.); taochunhuimail@163.com (C.T.); gsyeying@zju.edu.cn (Y.Y.); qiujinwangzju@163.com (Q.W.); zhouyifan0803@163.com (Y.Z.); 15757174920@163.com (Q.J.); 2 Key Laboratory of Submarine Geosciences, State Oceanic Administration, Hangzhou 310012, China; cw@sio.org.cn; 3 Second Institute of Oceanography, Ministry of Natural Resources, Hangzhou 310012, China; 4 Shandong SEI Science & Technology Co., Ltd., Jinan 250002, China; huangyuanfeng121@163.com

**Keywords:** ion-selective electrode, silicate, potentiometric, pH response, sensitivity

## Abstract

Ion-Selective Electrode (ISE) is an emerging technology for in situ monitoring of the chemical concentrations of an aqueous environment. In this work, we reported a novel all-solid-state silicate ISE, using an Ag/Pb/PbSiO_3_ electrode. This electrode responded to aqueous SiO_3_^2−^ with a reasonable slope of −31.34 mV/decade and a good reproductivity. The linear range covered from 10^−5^ M to 10^−1^ M, for the Na_2_SiO_3_ solutions and the response time was generally less than 5 s. Its potentiometric response to pH and silicate indicated that the prepared electrode was sensitive to silicate, rather than pH. Compared to the traditional liquid ISE, our all-solid-state silicate electrode was resistant to high pressure and could be used in situ, in deep water. In addition, the miniaturized electrodes (diameter of 0.4 mm and a length of 2–3 cm) could be easily integrated into a multi-modal sensor, which could simultaneously determine multiple parameters. Our prepared silicate ISE could potentially be used to determine the presence of silicate in a low-chloride aqueous environment, where the ISE exhibited better selectivity for silicate, over interfering ions such as, SO_4_^2^^−^, NO_3_^−^, CH_3_COO^−^, CO_3_^2^^−^, and PO_4_^3^^−^.

## 1. Introduction

As one of the major forms of silicon in the aqueous environment, silicate (SiO_3_^2−^) is an essential component for hard body parts and for outer skeletons of aquatic lives. It also acts as an essential nutrient for the growth of marine organisms [1]. When the silicate concentration exceeds a certain level, however, it will trigger eutrophication [2]. Therefore, it is important to determine the silicate concentrations in seawater or freshwater, which could help better understand the distribution, bio-availability, transportation, and global cycling of silicon [3]. Commonly used methods to determine the silicate concentration mostly depend on laboratory analysis with instruments such as, inductively-coupled plasma atomic emission spectrometry (ICP-AES) [4], spectrophotometry [5,6], and the silicon molybdenum blue method [7,8,9,10,11]. These methods, however, require sample pretreatment and in situ or continuous monitoring of the silicate concentration. All-solid-state ion selective electrode (ISE) is an emerging electrochemical technology for in situ determination of the chemical concentrations of aqueous environment, with advantages of high sensitivity, rapid response, and simple operation [12]. It could continuously monitor the aqueous solution, which could change with environmental factors such as climate, temperature, and pressure [13].

Previous researchers have successfully fabricated all-solid-state ISEs for the determination of CO_3_^2^^−^ [14], PO_4_^3^^−^ [15], NO_3_^−^ [16], NH_4_^+^ [17], K^+^ [18], Cd^2+^ [19], and Ca^2+^ [20,21]. Nevertheless, an all-solid-state ISE for silicate has not yet been reported, to our best knowledge. In this study, we fabricated a novel all-solid-state Ag/Pb/PbSiO_3_ ISE, using an Ag wire as the substrate. A PbSiO_3_ film was used as the membrane, as well as the elective ion-to-electron transducer, which could selectively identify silicate in aqueous solutions. Scanning Electron Microscopy (SEM) coupled with Energy Disperse Spectroscopy (EDS) indicated that we have successfully prepared an Ag/Pb/PbSiO_3_ electrode with PbSiO_3_ particles (with diameters of 0.2–0.5 μm) non-uniformly distributed in the Pb film (Figures S1 and S2). The mechanism of the silicate ISE was governed by a reversible ionic and electron exchange between the membrane and the metal, which has been discussed in the supplementary information.

## 2. Preparation of the Silicate Electrode

In a typical procedure, an Ag wire with a diameter of 0.4 mm and a length of 2–3 cm, was polished by a chamois leather, doped with 0.05 mm alumina powders. Then it was cleaned by an ultrasonic cleaner KQ218 (Shumei Company, Shenzhen, China), for 10 min. An abrasive paper was used to polish the Pb wire with a diameter of 0.6 mm. The Ag wire was set as the anode and the Pb wire was set as the cathode, 5 wt% Pb(NO_3_)_2_ solution was used as the electrolyte. Pb film was formed and coated on the Ag wire, using a two-electrode system by a CHI660D electrochemical workstation (Chenhua Company, Shanghai, China). The coating process operated under a constant potential of +0.6 V for 50 s, at room temperature. Then, the Ag wire was further coated by a PbSiO_3_ sensitive membrane, using the CHI660D electrochemical workstation. The processing conditions were as follows. The Ag wire was coated by the Pb film, as the working electrode, a commercial Ag/AgCl (Ag/AgCl electrode in saturated KCl) electrode (Gaoss Union Electronic Technology Company, Wuhan, China) was used as the reference electrode, a platinum electrode (Gaoss Union Electronic Technology Company, Wuhan, China) was used as the auxiliary electrode, and 0.1 M Na_2_SiO_3_ solution was used as the electrolyte. The potential was kept as +0.8 V, and the coating lasted for 100 s. In a N_2_ gas environment, the silicate ISE (Ag wire coated by Pb and PbSiO_3_ films) was prepared, after heating in a temperature-controlled furnace (Nabertherm GmbH, Lilienthal, Germany), at 110 °C, for 10 h.

## 3. Results and Discussion

### 3.1. Linear Range, Response Time, and Reproductivity of the Silicate ISE

**Figure 1:**
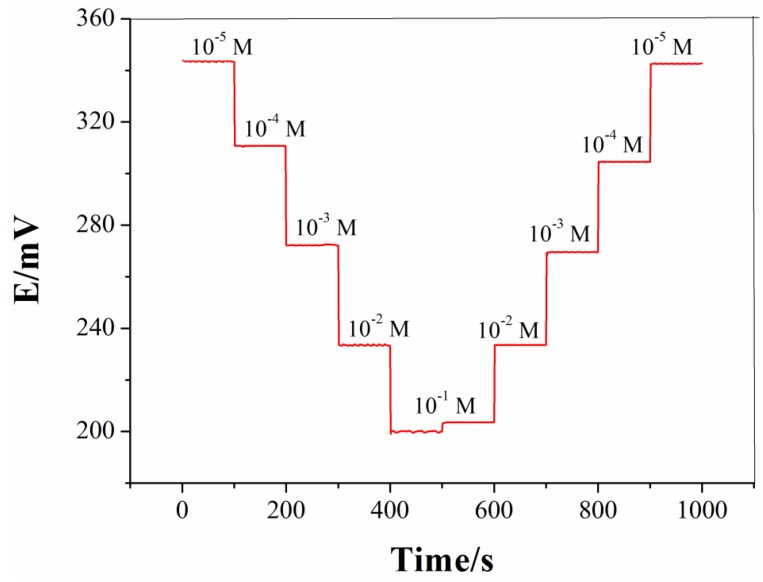
The calibrated curve of the silicate Ion-Selective Electrode (ISE) for Na_2_SiO_3_ solutions with concentrations ranging from 10^−5^ M to 10^−1^ M.

Figure 1 shows the calibrated curve of an all-solid-state silicate ISE in Na_2_SiO_3_ solutions, with concentrations ranging from 10^−5^ to 10^−1^ M. The potentials were 343.3 mV, 310.7 mV, 272.2 mV, 233.5 mV, and 200.1 mV from 10^−1^ M to 10^−5^ M, and then increased from 203.5 mV, 233.6 mV, 269.6 mV, 304.6 mV, to 342.5 mV from 10^−5^ M to 10^−1^ M. The prepared silicate ISE exhibited stable responses for all studied Na_2_SiO_3_ solutions, suggesting that the sensor held a linear range of 10^−5^–10^−1^ M. In addition, the comparable potentials at the same concentrations indicated a good repeatability of the prepared ISE.

**Figure 2:**
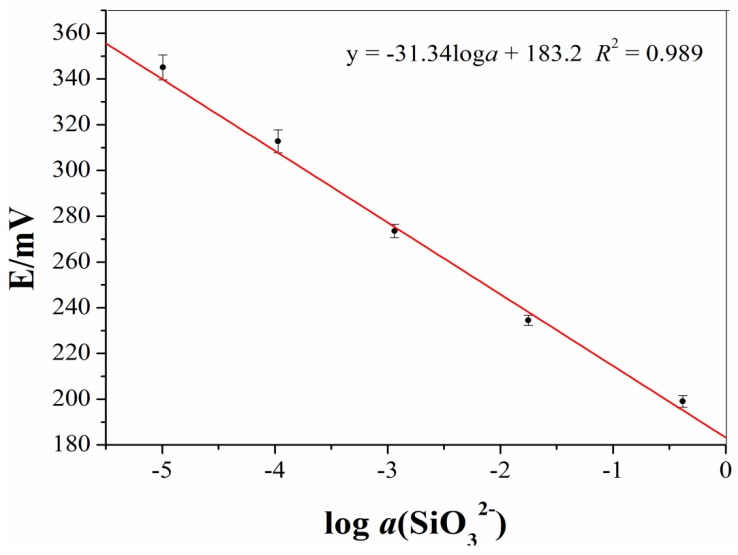
The calibration curves of the seven all-solid-state silicate ISEs that were prepared using the same method.

We prepared seven silicate electrodes, using the same method, to check the reproductivity of our preparation method. As shown in Figure 2, the measurement errors gradually increased with a decreasing Na_2_SiO_3_ solution concentration, which might be ascribed to two reasons: (1) The errors generated during the preparation of solutions, where a lower concentration usually produced larger errors; and (2) the sensitivity of the electrode decreased with a decreasing concentration [14,16]. Nevertheless, the calculated slope of the average potentials was −31.34 mV/decade, which was close to the theoretical value for divalent ions (−29.58 mV/decade) [22]. Furthermore, the correlation coefficients of 0.989 suggested that the preparation method was reproductive and the prepared ISE could effectively determine the silicate concentrations of aqueous solutions.

The response time of an electrode, measures the rate of the potentiometric response to achieve a steady value. It is an important analytical parameter since it determines the throughput of the sensor [23]. In this study, we immersed the electrode into Na_2_SiO_3_ solutions, with concentrations of 10^−2^ and 10^−3^ M, to study its response time for silicates (Figure S3). The potential was 268.4 mV, when the silicate electrode was put into the 10^−3^ M Na_2_SiO_3_ solution. Then the potential slowly increased and fluctuated with time. After about 2 s, the potential was stabilized at 273.5 mV. In a 10^−2^ M Na_2_SiO_3_ solution, the potential was started as 231.8 mV, then gradually changed to 236.0 mV within 5 s, and finally fluctuated at about 236.2 mV. These results suggested we could obtain reasonable signals in less than 5 s. This value was longer than that of an all-solid-state NH_4_^+^ electrode (0.5–2 s) [17], but was less than that of an all-solid-state NO_3_^−^ electrode (within 10 s) [16]. In addition, some reported ISEs required tens of seconds or even several minutes to reach a stable response [24]. 

### 3.2. pH Response of the Prepared Silicate ISE

**Figure 3:**
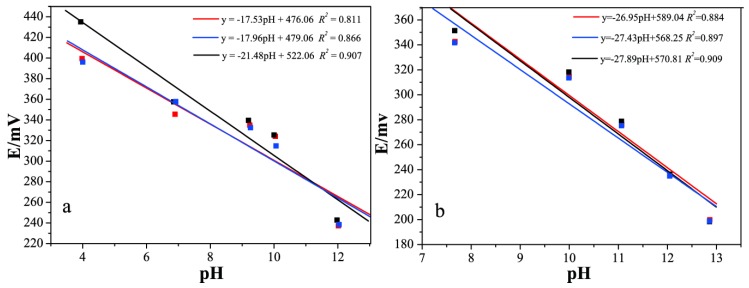
The correlation of the prepared electrode (**a**) the response potential and the pH values of standard pH buffers, and (**b**) the response potential and the pH values of the Na_2_SiO_3_ solutions with concentrations of 10^−1^–10^−^^5^ M.

Silicate in an aqueous solution will hydrolyze to form OH^−^, which subsequently changes the pH value of the solution. Therefore, it was important to study the pH response of our silicate ISEs. In this study, we examined the response of the silicate ISE to pH, both with and without silicate ions. We used standard pH buffers (pH = 12.000, 10.000, 9.182, 6.864, and 4.003) as the pH solutions. Three silicate electrodes were immersed in the standard pH solutions to record the potentiometric response. As shown in Figure 3a, the slopes of the fitted lines were 17.53 mV/pH, 17.96 mV/pH, and 21.48 mV/pH, respectively, which dramatically deviated from the theoretical values for monovalent ions (59.16 mV/pH). This indicated that the pH response of our prepared silicate sensor could not be explained by the Nernst Law [25]. Additionally, the relatively low correlation coefficients (*R*^2^ of <0.91) also suggested that our silicate ISE did not show a reasonable potentiometric response to the pH values.

**Figure 4:**
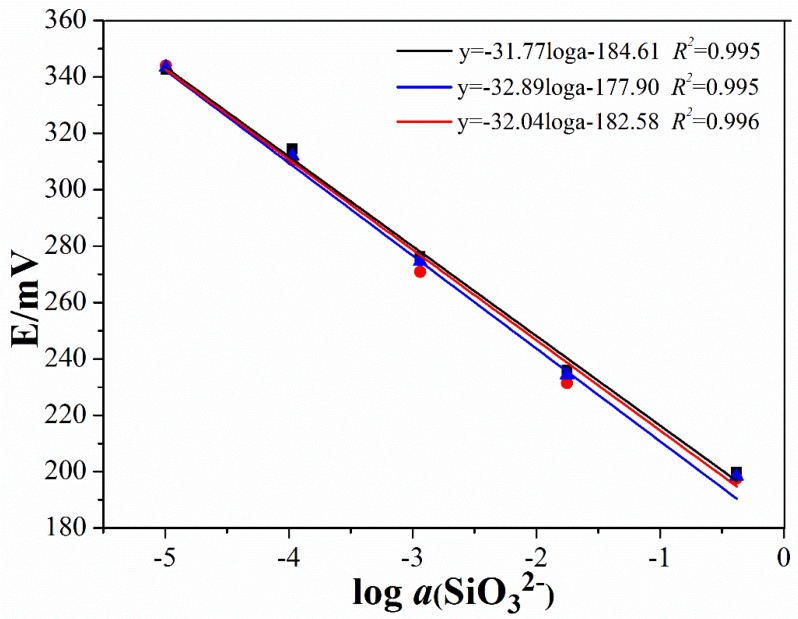
The correlation between the response potentials of the three all-solid-state silicate ISEs and the activity of the Na_2_SiO_3_ solutions with concentrations of 10^−1^–10^−5^ M.

Furthermore, we determined the pH values of 10^−1^–10^−5^ M Na_2_SiO_3_ solutions, by a Mettler Toledo InLab Expert Pro-ISM-IP6 (ME) pH glass electrode (Kuosi, Shanghai, China). All Na_2_SiO_3_ solutions with different concentrations exhibited pH values > 7, due to the hydrolysis of the SiO_3_^2^^−^. With the exponential increase of the Na_2_SiO_3_ concentrations from 10^−5^ M to 10^−1^ M, the pH values of the solutions increased from 7.669 to 9.996, 11.069, 12.053, and finally to 12.864. The calculated slopes of the potential–the pH correlations (Figure 3b)—were 26.95 mV/pH, 27.43 mV/pH, and 27.89 mV/pH, for the three silicate ISEs that were prepared using the same method. These values also significantly differed from the theoretical values of the H^+^ (59.16 mV/pH). Additionally, the low correlation coefficients again confirmed that the pH response of our silicate ISE was unreasonable. The correlations between the response potential and the activity of the Na_2_SiO_3_ solutions (Figure 4), on the contrary, exhibited near Nernstian slopes from −31.77 to −32.04 mV/decade and an *R*^2^ > 0.99. It suggests that the response of our prepared electrode to silicate ions was linear and stable. In conclusion, these results indicated the potentiometric response of our silicate ISE in different Na_2_SiO_3_ solutions, due to the silicate ions, rather than the pH values.

### 3.3. Selectivity 

An important property of an ISE is the selectivity of the primary ion over interfering ions. The selectivity determines whether the sensor could be utilized in realistic samples [26]. The fixed interference method (FIM) is a classic method to calculate the selectivity of the electrode. The selectivity coefficients (*K_i,j_*) was calculated on the basis of the following equation:

(1) Ki,j=ai(aj)zizj

where

*a_i_* = lower detection limit of primary ions when interfering ions existed
*a_j_* = activity of the interfering ions
*z_i_* = charge of the primary ions
*z_j_* = charge of the interfering ions

Selectivity factors log*K_i__,j_* < 0 indicates a preference for measuring ion *i*, relative to the interfering ion *j.* The smaller the log*K_i__,j_* values, the better the selectivity of the electrode for primary ions [27]. In this work, we studied the selectivity of the prepared ISE between SiO_3_^2^^−^ and NO_3_^−^, SO_4_^2^^−^, CH_3_COO^−^, Cl^−^, CO_3_^2^^−^, or PO_4_^3^^−^. The concentrations of the interfering ions were fixed at 10^−3^ M to estimate the selectivity coefficients. As shown in Table 1, the log*K_i__,j_* values were < 0 for all interfering ions, except for Cl^−^, indicating that the prepared electrode exhibited a good selectivity over the other ions. Nevertheless, CH_3_COO^−^ and CO_3_^2^^−^ would hydrolyze in aqueous solutions (Equation (2)–(4)) to produce OH^−^ ions and, consequently, would affect the pH value of the solution. Therefore, the CH_3_COO^−^ and the CO_3_^2^^−^ were potential interfering ions (log*K_i__,j_* = −0.21 and −0.53). The main potential interfering ion was Cl^−^; its log*K_i__,j_* achieved a value of 1.11 and the calculated slope was −23.43, which differed, dramatically, from the theoretical value. It was indicated that our prepared silicate ISE was not suitable for a high Cl^−^ environment. Additionally, the production of insoluble AgCl (*K_sp_*(AgCl) = 1.8 × 10^−10^), between the Ag wire of the silicate ISE and the Cl^−^ ions, in solution, might be another mechanism to create a Cl^−^ interference.

CH_3_COO^−^ + H_2_O ⇌ CH_3_COOH + OH^−^  *K* = 1.77 × 10^−5^ (2)

CO_3_^2−^ + H_2_O ⇌ HCO_3_^−^ + OH^−^    *K*_1_ = 2.13 × 10^−4^ (3)

HCO_3_^−^ + H_2_O ⇌ H_2_CO_3_ + OH^−^    *K*_2_ = 2.25 × 10^−8^ (4)

## 4. Conclusions

In summary, we fabricated a novel solid-state silicate ion-selective electrode by an electrochemical method. The prepared ISE showed a wide linear range (10^−5^ to 10^−1^ M) for Na_2_SiO_3_ solutions, and a fast response time of less than 5 s. The calculated calibration slope was about −31.34 mV/decade, comparable to the theoretical Nernstian slope for divalent ions. The pH response of the electrode indicated that the potentiometric response was caused by the silicate ions rather than pH values. Meanwhile, the prepared electrode showed a good selectivity towards silicate, over other ions, including SO_4_^2^^−^, NO_3_^−^, CH_3_COO^−^, CO_3_^2^^−^, and PO_4_^3^^−^. Nevertheless, the application of the prepared electrode was highly restricted to low Cl^−^ environment (such as freshwater), because the electrode suffered significant interference from Cl^−^. In our future work, we will modify the silicate electrode and try to eliminate the Cl^−^ interference.

## Figures and Tables

**Table 1:** Selectivity coefficients measured for silicates, by a fixed interference method.

Interfering Ions	Slope	*R* ^2^	log*K_i,j_*
NO_3_^−^	−32.38	0.99	−0.10
SO_4_^2−^	−32.38	0.98	−1.06
CH_3_COO^−^	−29.04	0.99	−0.21
Cl^−^	−23.43	0.93	1.11
CO_3_^2−^	−26.45	0.98	−0.53
PO_4_^3−^	−26.07	0.96	−1.38

## Supplementary Materials

The following are available online at http://www.mdpi.com/1424-8220/19/3/525/s1, Figure S1: SEM images of (**a**) Pb film on the surface of the Ag wire, (**b**) PbSiO_3_ membrane before heating at 110 °C, and (**c**,**d**) PbSiO_3_ membrane, after heating, at 110 °C, for 10 h. Figure S2: The Energy Disperse Spectroscopy (EDS) images of the silicate Ion-Selective Electrode (ISE). The wt% of Ag, O, Pb, and Si were 84.69%, 9.04%, 3.15%, and 3.12%, respectively. The presence of Pb, Si, and O in the samples indicated that we had prepared a Pb–Si–O substance, which was probably PbSiO_3_. Figure S3: The response time of the silicate ISE, in Na_2_SiO_3_ solutions with concentrations of 10^−2^ and 10^−3^ M.
