# Supplementary material for: An All-Solid-State Silicate Ion-Selective Electrode Using PbSiO3 as a Sensitive Membrane
Source: Sensors (Basel). 2019 Jan 27;19(3):525. doi: 10.3390/s19030525 (PMC6386971; doi:10.3390/s19030525)
Supplement: Supplementary file 1 [file sensors-19-00525-s001.pdf]

Supplementary Information

An All-Solid-State Silicate Ion-Selective Electrode Using $PbSiO_3$ as a Sensitive Membrane

Rongrong Wu [1,2,3], Xue-Gang Chen [1,*], Chunhui Tao [1,2,3], Yuanfeng Huang [4], Ying Ye [1], Qiujin Wang [1], Yifan Zhou [1], Quan Jin [1] and Wei Cai [2,3]

1 Ocean College, Zhejiang University, Zhoushan 316021, China; 18358087818@163.com (R.W.); taochunhuimail@163.com (C.T.); gsyeying@zju.edu.cn (Y.Y.) qiujinwangzju@163.com (Q.W.); zhouyifan0803@163.com (Y.Z.), 15757174920@163.com (Q.J.)
2 Key Laboratory of Submarine Geosciences, State Oceanic Administration, Hangzhou 310012, China; cw@sio.org.cn
3 Second Institute of Oceanography, Ministry of Natural Resources, Hangzhou 310012, China
4 Shandong SEI Science & Technology Co., Ltd. Jinan 250002, China; huangyuanfeng121@163.com
* Correspondence: chenxg83@zju.edu.cn

Received: 6 December 2018; Accepted: 22 January 2019; Published: 27 January 2019

1. Experiment

Materials

Sodium salts (sodium silicate, sulfate, acetate, chloride, nitrate, carbonate, phosphate, and lead nitrate) and standard pH buffers were obtained from Aladdin Chemicals (Shanghai, China). Ethanol was purchased from the Sinopharm Chemical Reagent Company (Shanghai, China). Ag wire (99.9%, 0.4 mm in diameter) and Pb wire (99.9%, 0.6 mm in diameter) was obtained from the Precious Materials Company of Changzhou, China. All materials were used without any further purification, and all solutions were prepared fresh.

Characterizations of the Prepared Silicate Electrode

The linear range, response time, and reproductivity of the ISE was evaluated in a series of Na_2SiO_3 solutions, at room temperature, under an open circuit. The pH response of the electrode was investigated by immersing the prepared ISE into aqueous solutions, with determined pH values. The selectivity of the ISE was known by the fixed interference method (FIM), where the prepared ISE was measured in a mixture of Na_2SiO_3 (10^{-1}–10^{-4} M) and other interference compounds. The studied interference ions included SO_4^{2-}, NO_3^-, CH_3COO^-, Cl^-, CO_3^{2-}, and PO_4^{3-}. The commercial Ag/AgCl (Ag/AgCl electrode in saturated KCl) reference electrode was used as the reference electrode, in all potentiometric measurements.

2. Scanning Electron Microscopy (SEM)

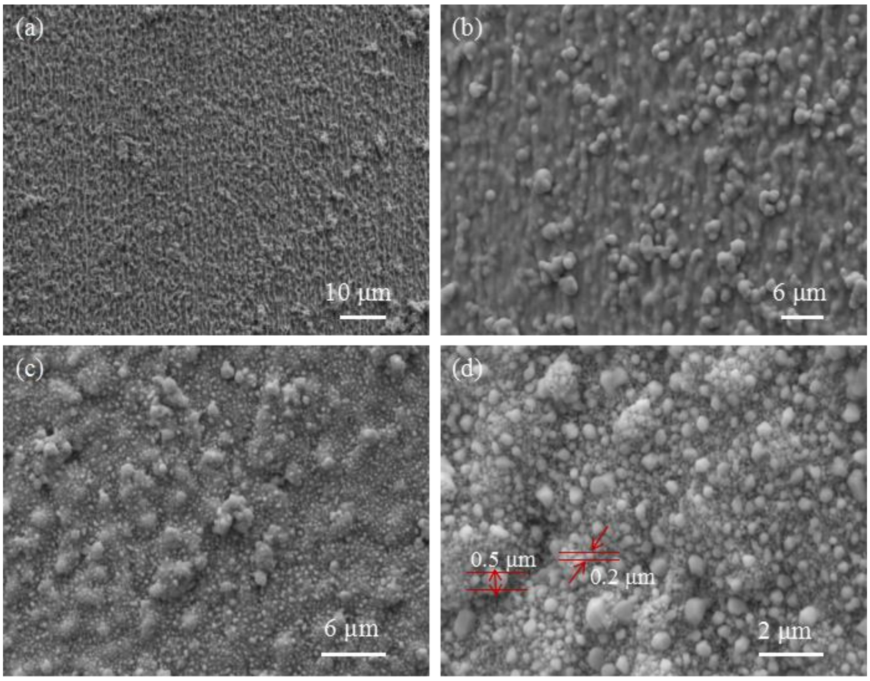

Figure S1. SEM images of (**a**) Pb film on the surface of the Ag wire, (**b**) $PbSiO_3$ membrane before heating at 110 °C, and (**c,d**) $PbSiO_3$ membrane, after heating, at 110 °C, for 10 h.

3. Energy Dispersive X-ray Spectroscopy (EDS).

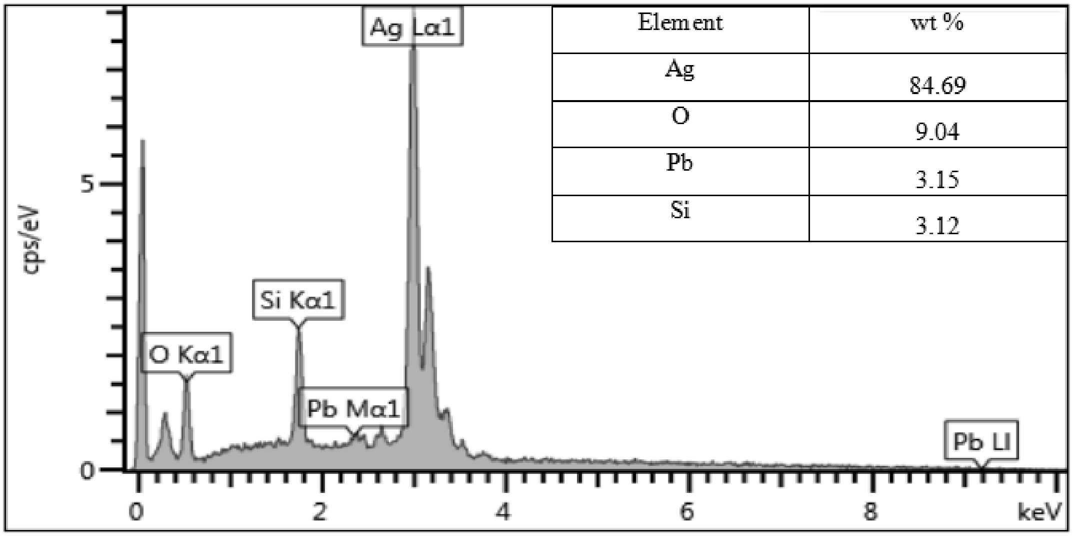

Figure S2. The Energy Disperse Spectroscopy (EDS) images of the silicate Ion-Selective Electrode (ISE). The wt% of Ag, O, Pb, and Si were 84.69%, 9.04%, 3.15%, and 3.12%, respectively. The presence of Pb, Si, and O in the samples indicated that we had prepared a Pb–Si–O substance, which was probably $PbSiO_3$.

4. The Mechanism of the Silicate ISE

An all-solid-state electrode with crystal membrane usually consists of a substrate and a sensitive membrane. The substrate should be conductive and stable (e.g., metal wire or glass carbon rod), while the membrane is usually an insoluble salt with a solubility below 10^{-6} M, which can exchange ions with solutions. As the all-solid-state electrode eliminates the internal

reference solution, the potential transfers through substrate–membrane interface, which is reversible between the ionic and electron response. The Ag/AgCl electrode is widely used a solid-state electrode that is governed by a reversible ionic and electron exchange between the membrane and metal. The insoluble salt should be directly in contact with the coated metal so that the metal wire can detect the response that is related to the concentration of the target ions. The mechanism of our prepared Ag/Pb/$PbSiO_3$ silicate electrode is similar to that of the Ag/AgCl chloride electrode. We used the Ag/Pb instead of the Pb as the substrate, because Pb is soft, easily broken, and is actively toxic. $PbSiO_3$, an insoluble silicate, was used as the sensitive membrane for responding to SiO_3^{2-}, in solution. The working mechanism was as follows. The SiO_3^{2-} ions in the solution equilibrated with SiO_3^{2-} of the sensitive membrane (Equation 1), while the Pb^{2+} of the membrane exchanged electrons with the Pb wire (Equation 2). In this process, the $PbSiO_3$ membrane, which also acts as the ion–to–electron transducer, converted the chemical signals into electrical signals, and produced the potentiometric responses. This process was reversible and, therefore, the prepared ISE could be used to monitor the silicate concentration of aqueous environments.

$$SiO_3^{2-} \text{ (membrane)} \rightleftharpoons SiO_3^{2-} \text{ (solution)} \quad (1)$$

$$Pb^{2+} \text{ (membrane)} \rightleftharpoons Pb \text{ (metal)} - 2e^- \quad (2)$$

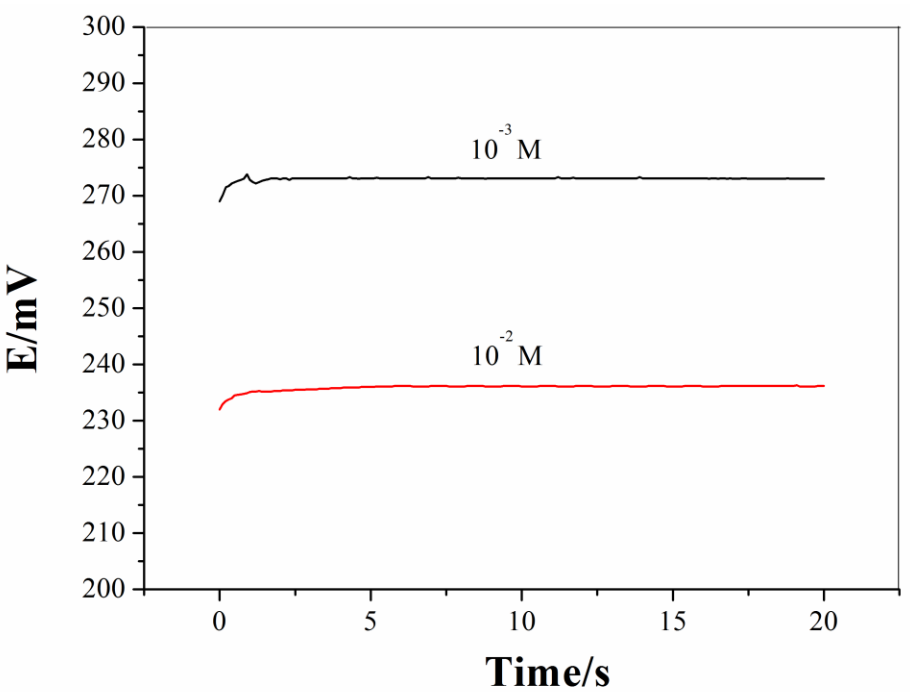

Figure S3. The response time of the silicate ISE, in Na_2SiO_3 solutions with concentrations of 10^{-2} and 10^{-3} M.
